# Daytime Restricted Feeding Affects Day–Night Variations in Mouse Cerebellar Proteome

**DOI:** 10.3389/fnmol.2021.613161

**Published:** 2021-04-12

**Authors:** Fabrice Bertile, Marine Plumel, Pauline Maes, Aurélie Hirschler, Etienne Challet

**Affiliations:** ^1^Institut Pluridisciplinaire Hubert Curien, LSMBO, Université de Strasbourg, Centre National de la Recherche Scientifique, Strasbourg, France; ^2^Institut des Neurosciences Cellulaires et Intégratives, Université de Strasbourg, Centre National de la Recherche Scientifique, Strasbourg, France

**Keywords:** food restriction, circadian rhythm, cerebellum, proteomics, circadian clock, neuroproteomics

## Abstract

The cerebellum harbors a circadian clock that can be shifted by scheduled mealtime and participates in behavioral anticipation of food access. Large-scale two-dimensional difference gel electrophoresis (2D-DIGE) combined with mass spectrometry was used to identify day–night variations in the cerebellar proteome of mice fed either during daytime or nighttime. Experimental conditions led to modified expression of 89 cerebellar proteins contained in 63 protein spots. Five and 33 spots were changed respectively by time-of-day or feeding conditions. Strikingly, several proteins of the heat-shock protein family (i.e., Hsp90aa1, 90ab1, 90b1, and Hspa2, 4, 5, 8, 9) were down-regulated in the cerebellum of daytime food-restricted mice. This was also the case for brain fatty acid protein (Fabp7) and enzymes involved in oxidative phosphorylation (Ndufs1) or folate metabolism (Aldh1l1). In contrast, aldolase C (Aldoc or zebrin II) and pyruvate carboxylase (Pc), two enzymes involved in carbohydrate metabolism, and vesicle-fusing ATPase (Nsf) were up-regulated during daytime restricted feeding, possibly reflecting increased neuronal activity. Significant feeding × time-of-day interactions were found for changes in the intensity of 20 spots. Guanine nucleotide-binding protein G(o) subunit alpha (Gnao1) was more expressed in the cerebellum before food access. Neuronal calcium-sensor proteins [i.e., parvalbumin (Pvalb) and visinin-like protein 1 (Vsnl1)] were inversely regulated in daytime food-restricted mice, compared to control mice fed at night. Furthermore, expression of three enzymes modulating the circadian clockwork, namely heterogeneous nuclear ribonucleoprotein K (Hnrnpk), serine/threonine-protein phosphatases 1 (Ppp1cc and Ppp1cb subunits) and 5 (Ppp5), was differentially altered by daytime restricted feeding. Besides cerebellar proteins affected only by feeding conditions or daily cues, specific changes in in protein abundance before food access may be related to behavioral anticipation of food access and/or feeding-induced shift of the cerebellar clockwork.

## Introduction

The main functions and behaviors, such as food intake and sleep, display circadian rhythms (i.e., endogenous rhythms close to 24 h). These rhythms are actually generated by internal clocks that allow cells, organs and the whole organism to anticipate and adapt to predictable changes in the environment. More specifically, circadian clocks depend on self-sustaining molecular oscillations that produce internal rhythms through the synthesis of target proteins, which deliver intracellular and, sometimes, extracellular time signals. In mammals, circadian clocks are organized into a multi-oscillating network that includes a master clock in the suprachiasmatic nuclei of the hypothalamus and many secondary clocks in the brain and peripheral organs (Fukuhara and Tosini, 2003; Golombek and Rosenstein, 2010; Bechtold and Loudon, 2013). The cerebellum harbors a circadian clock, as evidenced notably by self-sustained oscillations of clock proteins in organotypic slices of mouse cerebellum (Mendoza et al., 2010). Contrary to what happens in suprachiasmatic cells of the master clock (Brown and Piggins, 2007), the cerebellar clock does not appear to regulate rhythmic properties of neuronal membranes (Mordel et al., 2013). The role of this hindbrain clock for cerebellar function is not fully elucidated yet, although it may modulate efficiency of motor coordination on a daily basis. At least two challenging conditions can affect the cerebellar clock or its components. First, expression of the clock protein PER2 in the cerebellum can transiently increase in response to acute sleep deprivation (Curie et al., 2015), indicating that the cerebellum is sensitive to acute changes in vigilance states or their metabolic consequences. Secondly, scheduled meal times at odd times (i.e., food access limited to daytime, corresponding to the resting period in nocturnal mice and rats) can shift the cerebellar clockwork (Mendoza et al., 2010; Delezie et al., 2016), indicating that circadian oscillations in the cerebellum are sensitive to the synchronizing effects of meal time.

Behavioral anticipation of meal time, or so-called “food-anticipatory activity,” is a rhythmic behavior expressed by animals before scheduled access to food. Such a rhythmic behavior persists in the absence of a functional suprachiasmatic clock and displays circadian properties, leading to the concept of a “food clock” which would control temporal anticipation of meal time and daily availability of food (Mistlberger, 2011; Challet, 2019). The food clock is currently viewed as a multi-oscillating brain network involving secondary clocks in several structures of the mediobasal hypothalamus and the brainstem in connection with the striatum and the cerebellum (Davidson, 2009; Mendoza et al., 2010; Mistlberger, 2011; Gallardo et al., 2014). Several experimental findings support an implication of the cerebellum in the food clock. First, food-anticipatory activity is associated with increased expression of c*-Fos* and decreased glucose utilization in the cerebellar cortex (de Vasconcelos et al., 2006; Poulin and Timofeeva, 2008). Secondly, genetic impairment (i.e., *hot-foot* mutation) or immunotoxical alteration (i.e., OX7-saporin treatment) of cerebellar function leads to huge reduction of meal anticipation (Mendoza et al., 2010). Thirdly, dampened food-anticipatory in mice with brain deletion of the clock gene *Rev-erb*α is concomitant with impaired transcriptional changes in the cerebellum (Delezie et al., 2016). Furthermore, conditional deletion of Bmal1 in the granule cells of the cerebellum does not impair food-anticipatory activity (Bering et al., 2017). This indicates that if the cerebellar clock plays some role in this specific behavior, the clockwork of other cell types, such as Purkinje cells (Mendoza et al., 2010), may be involved.

The mechanisms by which the food clock controls meal anticipation and how timed restricted feeding can phase-shift the circadian clock in the cerebellum are still poorly understood. Our previous study has demonstrated that the circadian landscape of cerebellar proteome in mice fed *ad libitum* is bimodal with two daily peaks of rhythmically expressed proteins, clustered in the middle of the rest (i.e., midday) or active phases (i.e., midnight) (Plumel et al., 2019). The present study aims at characterizing day–night changes in cerebellar proteome by targeting these two time-points in mice fed during daytime or nighttime. More specifically, we wanted to determine which cerebellar proteins would be modulated by circadian or daily cues (independent of meal time: effect of time-of-day only), by homeostatic processes linked to food availability (effect of feeding conditions only), or by a combination of both factors (dependent on meal time: feeding × time-of-day interaction). Behavioral anticipation of meal time in food-restricted mice is expected to trigger specific changes before food access.

## Materials and Methods

### Animals and Study Design

Male C57BL6J, 3-month-old mice (Janvier labs, Le Genest-Saint-Isle, France) were individually housed in transparent plastic cages equipped with a running wheel (12.5 cm in diameter) in a temperature-controlled room (22 ± 1°C) under 12 h light and 12 h dark (LD 12:12) conditions, with lights on at 07:00 AM and lights off at 07:00 PM defining respectively Zeitgeber time (ZT) 0 and ZT12 (Chronobiotron platform, UMS 3415, CNRS and University of Strasbourg). Regular chow (SAFE 105, Augy, France) and water were provided *ad libitum*, except during the periods of restricted feeding. Access to food during restricted feeding was set automatically by the Fasting Plan system (Intellibio, Seichamps, France). Mice (*n* = 22) were fed *ad libitum* for 2 weeks. Thereafter, half of animals [restricted feeding (RF) group, *n* = 12] was exposed to daytime restricted feeding schedules in which food availability was progressively reduced to 6 h per day, from ZT6 to ZT12 for up to 2 weeks, as previously reported (Delezie et al., 2016). The other half [Control (Co) group, *n* = 10] remained with free access to food, except on the last day during which food was only available at night (see Supplementary Figure 1). On the day of sampling, half of each group was injected i.p. with a lethal dose of pentobarbital (200 mg/kg; CEVA, Libourne, France) either 5 h after lights on (ZT5; *n* = 6 RF; *n* = 5 Co), or 12 h later (i.e., 5 h after lights off, ZT17; *n* = 6 RF; *n* = 5 Co). Cerebellum was harvested and immediately frozen in liquid nitrogen and stored at –80°C. One control sample at ZT5 was lost, leading to a final *n* = 4 Co.

### Actimetry

A PC-based acquisition system (Vitalview, MiniMitter, Sunriver, OR, United States) recorded wheel-running activity 24 h a day. Daily rhythm of wheel-running activity was analyzed using a Clocklab software (Actimetrics, Evanston, IL, United States) associated to MatLab (MathWorks, France). Mean profiles of locomotor activity were quantified every 1 h during the last 7 days of recording.

### Cerebellum Proteomics Analysis

Unless otherwise specified, all reagents and chemicals were purchased from Sigma Diagnostics (St. Louis, MO, United States). Proteins were extracted from half of each cerebellum divided in the midline for two-dimensional difference gel electrophoresis (2D-DIGE) analysis. Briefly, cerebellar samples were incubated in a denaturing buffer (8 M urea, 2 M thiourea, 4% Chaps, 1% DTT, 0.5% Triton X-100, 0.005% TLCK, 0.02–2 mM protease inhibitors) for 1 h30. Samples were sonicated on ice (10–30 s, 135 W), then centrifuged (5 min, 12,000 × *g*, 4°C) to remove cell debris. Proteins were precipitated using trichloroacetic acid, pelleted by centrifugation (15 min, 14,000 × *g*, 4°C), and dissolved [7 M Urea, 2 M thiourea, 30 mM Tris (pH 8.5) and 4% CHAPS] after vacuum-drying (Speedvac, Thermo Fisher Scientific; Rockford, IL, United States). After sonication on ice (10 s, 135 W), protein concentrations were determined using the Bio-Rad Protein Assay (Bio-Rad, Hercules, CA, United States). Similarity of protein profiles between all samples was then checked on 10% SDS-PAGE acrylamide gels (10 μg loaded; 50 V for 90 min and then 100 V to complete migration) using Coomassie blue (Fluka, Buchs, Switzerland) staining.

Protein labeling was performed using a CyDye DIGE Fluor Minimal Dye Labeling Kit (GE HealthCare, Uppsala, Sweden). After reconstitution of CyDyes in anhydrous N,N-dimethylformamide, 50 μg of protein samples from the different groups were labeled using 400 pmol of either Cy3 and Cy5, and a mixture of all the samples consisting of equal amounts from each protein extract was labeled using 7.2 nmol of Cy2. This mixture sample was used afterward as an internal standard. Protein labeling was performed on ice for 30 min in the dark, then addition of 10 mM lysine allowed quenching of protein labeling (10 min in the dark on ice).

Prior to 2D gel electrophoresis, 50 μg of Cy2, Cy3, and Cy5-labeled protein samples were mixed to enable multiplexing, which was randomly performed to avoid any bias. Dilution of mixed samples was performed using 400 μl of electrophoresis buffer (7 M urea, 2 M thiourea, 2% CHAPS, 2% DTT, 2% ampholytes [SERVALYT Carrier Ampholytes 3–10, Serva, Heidelberg, Germany], a trace of bromophenol blue). Non-linear immobilized pH gradient strips (IPG Ready strip, 24 cm, pH 3–10, Bio-Rad, Hercules, CA, United States) were used for protein loading, and passive rehydration was performed by incubation for 1 h in the dark. A Protean Isoelectric focusing (IEF) cell (Bio-Rad, Hercules, CA, United States) was then used for active rehydration (50 V overnight). IEF was performed using voltage gradient steps (from 0 to 200 V in 1 h, from 200 to 1,000 V in 4 h, from 1,000 to 5,000 V in 16 h, then 5,000 V for 9 h), with a total focusing time of 95,500 Vh. After reduction (DTT buffer, 15 min, Serva) and alkylation (iodoacetamide buffer, 15 min, Serva) of proteins, electrophoresis was carried out using 12.5% polyacrylamide SDS-PAGE precast gels (2D HPE large gel NF 12.5% kits), and application of intensity steps (7 mA per gel for 30 min, 13 mA for 30 min, 20 mA for 10 min, 40 mA for 3 h50, 45 mA for 40 min) was performed using a HPE Flap Top Tower (Gel company, San Francisco, CA, United States).

After electrophoresis, gels were washed using water, and gel image acquisition was performed using an Ettan DIGE Imager (GE HealthCare) at 100 μm resolution. Progenesis Samespots analysis software (v4.5; Non-linear Dynamics) was used to analyse gel images, with minor corrections having been applied manually for consistency in spot matching. Image background was subtracted, then Cy3 and Cy5 spot intensities were normalized to the intensity of corresponding Cy2 spots. The ZT5 Co group was afterward chosen as the control, for which mean spot intensities were set to 1. Spot intensities in all other groups were then normalized to this control.

Differentially intense spots among mice groups were identified using statistical analysis (see below), then excised using a screen picker and One touch spot picker device (Proteomics Consult, Belgium). In-gel reduction and alkylation of proteins (Massprep Station, Waters, MicroMass, Manchester, United Kingdom) were performed as previously described (Plumel et al., 2014), from pooling of four gel spots that were nicely matched among four different gels. Proteins were in-gel digested using trypsin (Promega, Madison, WI, United States) for 12 h at 37°C. Tryptic peptides extraction was performed using 20 μL of 60% acetonitrile/0.1% of formic acid (Carlo Erba, Val de Reuil, France). Samples were dried using a speedvac prior to nanoLC-MS/MS analyses.

For peptide analysis, we used a nanoUPLC (NanoAcquityUPLC, Waters) system coupled to a hybrid mass spectrometer, either MaXis Q-Tof (Bruker Daltonics) or Synapt HDMS G1 Q-Tof (Waters, Milford, MA, United States) equipped with a Z-spray ion source and a lock mass system. The chromatographic solvent system consisted of 0.1% HCOOH in water (solvent A) and 0.1% formic acid in acetonitrile (solvent B). Concentration/desalting of 3μl of sample was performed using a trap column (C18, 180 μm × 20 mm, 5 μm; Waters) at 1% of B at a flow rate of 5 μl/min for 3 min. Afterward, peptide elution was performed using a separation column (BEH130 C18, 75 μm × 250 mm, 1.7 μm; Waters) maintained at 60°C and a 9 min gradient from 1 to 35% of B at a flow rate of 450 nL/min.

Mass spectrometers were operated in positive mode, with automatic switching between MS and MS/MS scans. The source temperature of the MaXis was set to 160°C with a spray voltage of −4.5 kV and dry gas flow rate of 5 L/min. Full scan MS spectra were acquired within a mass range of 100–2,500 m/z. Tuning Mix (Agilent Technologies, Paolo Alto, United States) was used for external mass calibration (mass range: 322–2,722 m/z) of the Tof (MaXis) before each set of analyses, and recalibration of acquired spectra to the applied lock mass [hexakis (2,2,3,3,-tetrafluoropropoxy)phosphazine; [M + H] + 922.0098 m/z] allowed for mass correction. MS acquisition time was set to 0.4 s, and MS/MS acquisition time to a range from 0.05 s (intensity > 2,50,000) to 1.25 s (intensity < 5,000). After acquisition of two MS/MS spectra for a given ion, this ion was excluded for 1 min. Up to five most intense multiply charged precursors per MS scan were isolated, using an isolation window adapted to the isolated m/z (2–5 m/z), then fragmented using energy collisional dissociation. This system was fully controlled by compass HyStar v3.2 and OtofControl v3.2 (Bruker Daltonics, GE).

For the Synapt G1, voltages were parametrized as follows: capillary voltage set to 3.5 kV, sample cone voltage to 35 V, and extraction cone voltage to 4.0 V. Calibration of the TOF was performed using Glu-fibrino-peptide B on the 50–2,000 m/z range, then it was corrected online with Glu-fibrinopeptide B as the lock-mass (MS calibrating ion (M + 2H)2 + at m/z 785.8426; MS/MS calibrating ion (M + H) + at m/z 684.3469). To this end, a lock spray interface was used and lock spray frequency was set to 30 s. Automatic switching between MS and MS/MS modes was used. The three most abundant peptides (intensity threshold: 40 count s^–1^; ions with 2 or 3 charges), were selected on each MS spectrum, then isolated and fragmented using collision-induced dissociation (CID). Argon was used as collision gas, and 2 different collisional energies were set up (from m/z 300 to 500: 14 and 18 eV; from m/z 501 to 600: 19 and 24 eV; from m/z 601 to 700: 24 and 28 eV; from m/z 701 to 800: 28 and 32 eV; from m/z 801 to 900: 32 and 39 eV; from m/z 901 to 1,000: 39 and 45 eV; from m/z 1,001 to 1,200: 45 and 55 eV; from m/z 1,201 to 1,700: 55 and 60 eV). Both MS (scan time: 0.5 s) and MS/MS (scan time: 0.7 s) scan ranges were set to m/z 100 to 2,500. The system was fully controlled by the MassLynx software (v.4.1., Waters).

Mascot^TM^ search engine (v2.6.2, Matrix Science, London, United Kingdom) was used to analyse MS/MS data. MSDA software suite (Carapito et al., 2014) was used to generate a target-decoy version of the *Mus musculus* protein database (Swissprot, April 2019, 17,007 target sequences) containing sequences for common contaminants (e.g., trypsin and keratins; 118 entries). Mascot search parameters were set as follows: MS and MS/MS tolerances of respectively 10 ppm and 0.05 Da (MaXis) or 25 ppm and 0.07 Da (Synapt), one missed cleavage tolerated, carbamidomethylation of cysteines set as fixed modification, and oxidation of methionines and acetylation of protein N-termini set as variable modifications.

Proline Studio software (v2.0)^1^ was used to apply stringent filtering criteria and retain high confidence identifications (Mascot peptide ion score > 25; peptide FDR < 1% based on e-values, protein FDR < 1% based on Mascot Modified Mudpit Scoring, minimum length of seven amino acids). Single peptide-based identifications and the identification of common contaminants, such as keratin and trypsin, were not considered. The mass spectrometry proteomics data have been deposited to the ProteomeXchange Consortium via the PRIDE partner repository with the dataset identifier PXD021056.

Because several proteins are often identified in a same gel spot, we used a “peptide counting” strategy to identify the major ones, i.e., the more abundant ones that were expected to have the main impact on spot intensity. This was done assuming that the higher the number of peptides assigned to a given protein, the more abundant this protein is. Hence, we compared the theoretical number of detectable tryptic peptides to the experimental number we identified. Theoretical tryptic peptides were predicted by considering that trypsin does not cleaves proteins when the tryptic site is followed by proline residue, by considering the possibility for missed cleavages and taking into account the adequate size of peptides for their detection by mass spectrometry (i.e., peptides of 7–39 amino acids based on our experimental data). We could determine that the theoretical number of detectable tryptic peptides for major and minor proteins in given gel spots was very similar (mean ratio major/minor = 1.1 ± 0.4). However, the fraction of detected over theoretical peptides was 4.4 ± 2.2 times greater for major than minor proteins with 4.8 ± 2.7 times more peptides identified for major versus minor proteins (16 ± 8 vs. only 4 ± 1). Major proteins were thereafter those that were considered to be responsible for variations in spot intensities, and minor ones were therefore not discussed.

Functional annotation enrichment analysis of proteomics data was performed in an unbiased fashion using the desktop version of DAVID (Ease v2.1) and an updated version of gene ontology (GO) and KEGG pathway databases (January 2021). Enriched GO terms and Kegg maps were filtered by only considering those with an Ease score lower than 0.1, a Benjamini *p*-value lower than 0.05, and a fold enrichment higher than 2. Enriched GO terms and Kegg maps were thereafter grouped together into broad functional categories, which were considered as enriched broad functions.

### Western-Blot Analyses

The second half of each cerebellum divided in the midline was used for protein extraction for Western blot analyses. Briefly, grinding was performed under liquid nitrogen (30 s at 30 Hz) using a Mixer Mill MM400 (Retsch, Eragny sur Oise, France). Proteins were afterward extracted using 10 μl of extraction buffer (8 M urea, 2 M thiourea, 2% CHAPS, 10 mM DTT, 30 mM Tris pH 8.8 and protease inhibitors) per mg of tissue. Samples were sonicated (10 s at 135 W) and incubated at 4°C under agitation for 90 min. Centrifugation was used to pellet and remove cell debris, then proteins were precipitated using trichloroacetic acid at 4°C, pelleted by centrifugation (15,000 × *g*, 15 min, 4°C), washed with cold acetone, and finally dissolved in Laemmli buffer. Total protein concentrations were determined using the Bradford Protein Assay kit from Bio-Rad.

Mini-PROTEAN TGX Stain-Free Precast Gels (8–16%, Bio-Rad, Hercules, CA, United States) were used to separate proteins from 5 μg of each sample. Gel images were acquired after activation using the Bio-Rad ChemiDoc Touch Imaging System and proteins were transferred to nitrocellulose membranes (0.2 μm) using the Bio-Rad Trans-Blot Turbo Transfer System. Blot images were immediately acquired, and, once protein transfer was confirmed, TBS-T (Tris 25 mM, NaCl 137 mM, KCl 2.68 mM, 5% Tween 20) containing 4% of BSA was used to block the membranes for 1 h at room temperature. Membrane incubation overnight at 4°C with primary antibodies was then performed. Primary antibodies against heat shock protein HSP 90-alpha (hsp90aa1; sc-13119, Santa Cruz Biotechnology, Dallas, TX, United States) and period circadian protein homolog 2 (PER2; Per21-A; Alpha Diagnostic International, San Antonio TX, United States) were diluted in the blocking solution at 1/1,000. Aryl hydrocarbon receptor nuclear translocator-like protein 1 (BMAL1; ab3350; Abcam, Paris, France) and 78 kDa glucose-regulated protein (GRP78, Hspa5; ab21685, Abcam) were diluted in the blocking solution at 1/200 and 1/500, respectively. Membranes were washed 3 times in TBS-T for 10 min, then incubated for 1 h at room temperature with a peroxidase-conjugated secondary antibody (Santa Cruz Biotechnologies) against IgG from rabbit (sc-2004) or mouse (sc-2005) diluted in the blocking solution at 1/5,000. Membranes were washed three times in TBS-T for 10 min, then incubated for 1 min in Luminata Classico Western HRP substrate (Merck Millipore, Molsheim, France). Chemiluminescent blot detection was achieved using the ChemiDoc Touch Imaging System (Bio-Rad), and blot images were analyzed using Bio-Rad Image Lab software v5.1. Signals were normalized to total proteins, as measured on the stain-free gel image. The ZT5 Co group was afterward chosen as the control, for which mean intensity values were set to 1. Blot signal intensities in all other groups were then normalized to this control.

### Statistical Analysis

Statistical analysis of wheel-running activity, proteomics quantitative data and western-blot analyses was performed under R software environment v3.4.0 (R Development Core Team, 2008). Normality distribution and homoscedasticity were checked using the Shapiro–Wilk test and the Bartlett test, respectively. For proteomics data, this was performed from log-transformed values. For body mass, food intake and wheel-running activity, the main effects of feeding conditions (daytime restricted feeding vs. control conditions: food *ad libitum* + nighttime feeding on day of sampling) and time (either beginning vs. end of experiment; and 24-h times-of-day) were tested by two-way analyses of variance (ANOVA) with repeated measures, followed by Tukey *post hoc* test. For proteomics and western-blot data, the effects of feeding conditions (daytime restricted feeding vs. control conditions: food *ad libitum* + nighttime feeding on day of sampling), times-of-day (ZT5 vs. ZT17), and their interactions were tested using two-way ANOVA. Pairwise comparisons were thereafter performed using the Tukey HSD *post hoc* test, which includes adjustment of *p*-values. The threshold for significance was set to *p* < 0.05.

## Results

**FIGURE 1:**
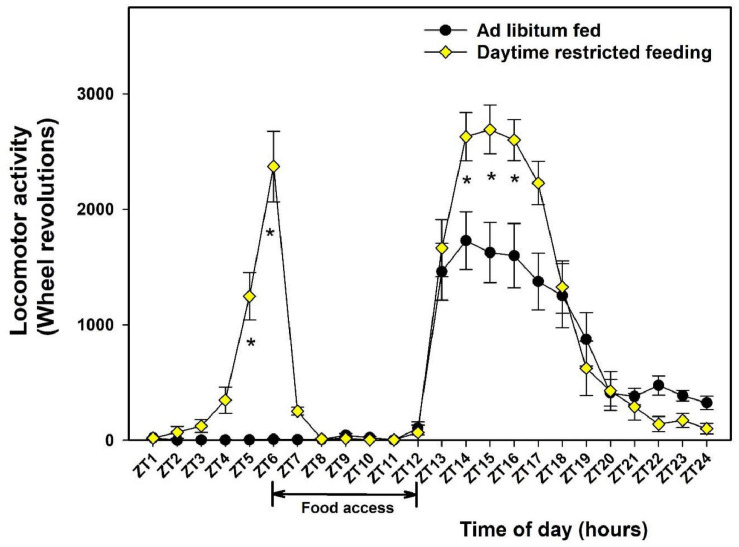
Daily profiles of wheel-running activity in control mice fed *ad libitum* (closed circles) or exposed to daytime restricted feeding (yellow diamonds). During daytime restricted feeding, food was available between Zeitgeber (ZT) 6 to ZT12 (see section “Materials and Methods”). For each individual, data were averaged during the last 7 days of recording before the day of sampling. During the analyzed period, control mice were fed *ad libitum*. *P < 0.05 between feeding conditions for a given ZT after *post hoc* Tukey comparison and two-way ANOVA with repeated measures.

Initial body mass of the mice was 23.2 ± 0.2 g. At the end of the study, the mass of control mice increased by 5.1 ± 0.5%, while that of food-restricted mice decreased by 7.5 ± 1.6% (main effect of feeding; *P* < 0.01). On average, the daily food intake in food-restricted mice was lower as compared to control mice (3.6 ± 0.1 vs. 4.2 ± 0.1 g, respectively; main effect of feeding; *P* < 0.01), representing a mild (i.e., 14%) reduction in calorie intake. As expected, control mice fed *ad libitum* displayed a nocturnal pattern of wheel-running activity starting after lights off (ZT12) and ending at lights on (ZT0; main effect of time-of-day, *P* < 0.01). To avoid any effect of daytime feeding on the sampling day, food access in this control group was restricted to nighttime (i.e., from ZT12 = lights off to ZT17). Mice challenged with a 6-h daytime restricted feeding (food access being from noon to lights off, that is, from ZT6 to ZT12) kept a main nocturnal pattern of locomotor activity, combined with increased activity in early night as compared to control mice (main effect of feeding conditions, *P* < 0.01). In addition, daytime-fed mice expressed a strong bout of wheel-running 2 h before meal access, corresponding to the so-called food-anticipatory activity (Figure 1).

To gain insights into cerebellum proteome changes, samples (*n* = 4–6/group) were analyzed individually (i.e., samples were not mixed or pooled) using 2D-DIGE-MS. On 2D-DIGE gels, 603 protein spots were nicely visualized and the intensity of 63 of them was found significantly changed (two-way ANOVA; *p*-value < 0.05; see Supplementary Figure 2 and Supplementary Table 1). Hence, the intensity of 37 protein spots was significantly changed due the feeding pattern only, that of 7 spots as a function of time-of-day only, and that of 13 spots only due to the interaction of the two variables. In addition, three spots were found modified due to both feeding pattern and time-of-day, two spots due to both time-of-day and interaction of the two variables, and only one due to the feeding pattern and interaction of the two variables.

**FIGURE 2:**
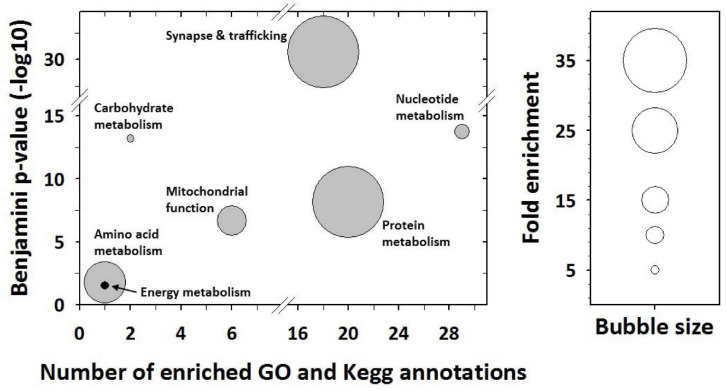
Functional annotation enrichment analysis from differentially expressed proteins. The analysis was performed using the desktop version of DAVID (Ease v2.1) from protein gene ontology and Kegg pathway annotations. Significantly enriched GO terms and Kegg pathway maps were determined using the following thresholds: Ease score < 0.1, Benjamini *p* value < 0.05, and fold enrichment > 2. Their grouping together allowed highlighting here enriched broad functions.

Mass spectrometry allowed 267 proteins to be identified in the 63 significant protein spots (see details in Supplementary Table 1). From the determination of most abundant (or “major”) proteins per spot (see section “Materials and Methods”), we finally retained 89 proteins whose abundance was changed significantly among mouse groups. Functional annotation analysis revealed that these differences between mice groups involve mostly proteins that are known to play a role in the following broad functions: synapse and trafficking, the metabolisms for nucleotides, carbohydrates, proteins and amino acids, and energy metabolism and the mitochondrial function (Figure 2).

### Effects of Time-of-Day on Cerebellar Proteins

**FIGURE 3:**
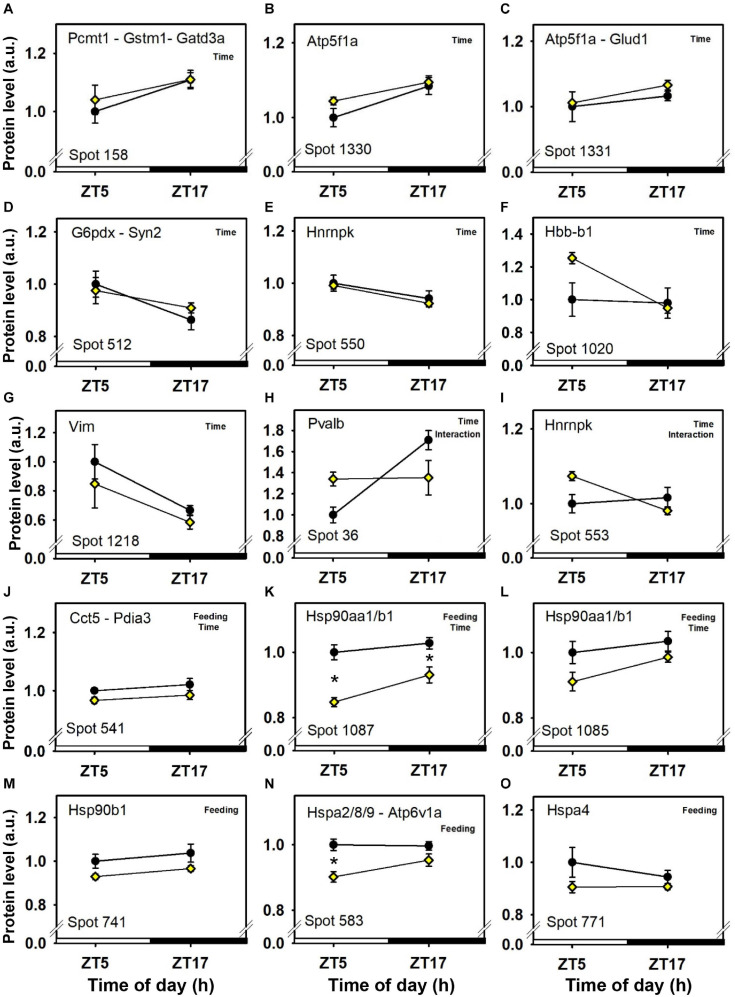
Day–night expression of cerebellar proteins mostly affected by time-of-day. From analyses in control mice fed *ad libitum* (closed circles) and mice exposed to daytime restricted feeding (yellow diamonds), “Time,” “Feeding,” and “Interaction” indicate significant (*P* < 0.05) main effects of time-of-day, feeding conditions or time-of-day × feeding interactions, respectively, as detected by two-way ANOVA (see section “Materials and Methods” for details). *P < 0.05 after *post hoc* Tukey comparison. Different part labels have been attributed to each differentially-expressed proteins; see details in the main text.

Seven cerebellar protein spots were affected only by time-of-day, independently of feeding time and without significant interaction (Figure 3 and Table 1). Three spots displayed day–night variations characterized by lower abundances of proteins at midday, including one redox regulator (glutathione S-transferase Mu 1, Gstm1; spot 158; Figure 3A) and mitochondrial proteins involved in the metabolism of glutamine (glutamine amidotransferase-like class 1 domain-containing protein 3A, Gatd3a; spot 158; Figure 3A, and glutamate dehydrogenase 1, Glud1; spot 1,331; Figure 3C), in cellular energy homeostasis (ATP synthase subunit alpha, Atp5f1a; spots 1,330 and 1,331; Figures 3B,C), and in protein repair notably against spontaneous deamidation [protein-L-isoaspartate(D-aspartate) O-methyltransferase, Pcmt1; spot 158; Figure 3A]. Instead, four other spots showing day–night variations showed higher protein abundances at midday, including key proteins for the pentose phosphate pathway (glucose-6-phosphate 1-dehydrogenase X, G6pdx; spot 512; Figure 3D), and for synapse activity (Synapsin-2, Syn2; spot 512; Figure 3D), cytoskeleton (Vimentin, Vim; spot 1,218; Figure 3G), and pre-mRNA processing (heterogeneous nuclear ribonucleoprotein K, Hnrnpk; spots 550 and 553; Figures 3E,I). Cerebellar levels of hemoglobin subunit beta-1 (Hbb-b1) were affected by time in one protein spot (spot 1,020; Figure 3F). Hbb-b1 was also detected in two other protein spots, thus reflecting the presence of isoforms possibly due to post-translational modifications (spots 1,019 and 1,022; see Figures 7G,H). From these two latter protein spots, statistical interactions were significant, Hbb-b1 levels were found to be higher at ZT5 in food-restricted mice, which may reflect higher amount of circulating red cells and/or increased blood flow.

**TABLE 1:** List of cerebellar proteins differentially expressed according to feeding conditions and/or time-of-day.

Spot N°	Protein name	Gene name	Subcellular location	Related functions	Effect	
537	RAC-beta serine/threonine-protein kinase	Akt2	Plasma membrane, nucleus, endosome	Metabolism	Feeding	Increased
279	Delta-aminolevulinic acid dehydratase	Alad	Cytosol, nucleus	Miscellaneous	Feeding	Increased
1,050	Cytosolic 10-formyltetrahydrofolate dehydrogenase	Aldh1l1	Cytoplasm	Metabolism	Feeding	Decreased
1,023	Fructose-bisphosphate aldolase C	Aldoc	Cytosol	Carbohydrate metabolism	Feeding	Increased
245	Annexin A5	Anxa5	Cytosol	Synapse and trafficking	Feeding	Decreased
453 + 454	ATP synthase subunit beta, mitochondrial	Atp5f1b	Mitochondrion	Mitoch. function, Energy metab.	Feeding	Decreased
583	V-type proton ATPase catalytic subunit A	Atp6v1a	Cytoplasm	Synapse and trafficking	Feeding	Decreased
493	V-type proton ATPase subunit B, brain isoform	Atp6v1b2	Membrane	Synapse and trafficking	Feeding	Decreased
1,244	Carbonic anhydrase-related protein	Ca8	Cytoplasm	Miscellaneous	Feeding	Decreased
536	Calreticulin	Calr	Endoplasmic reticulum, cytosol	Protein metabolism	Feeding	Decreased
544 + 1,145	T-complex protein 1 subunit zeta	Cct6a	Cytoplasm	Protein metabolism	Feeding	Decreased
29	Cytochrome c oxidase subunit 5B, mitochondrial	Cox5b	Mitochondrion	Mitoch. function, Energy metab.	Feeding	Increased
544	Dihydropyrimidinase-related protein 1	Crmp1	Cytoplasm, cytoskeleton	Synapse and trafficking	Feeding	Decreased
21	D-dopachrome decarboxylase	Ddt	Cytoplasm	Miscellaneous	Feeding	Increased
620	ATP-dependent RNA helicase DDX3X	Ddx3x	Nucleus, cytosol, membrane	Nucleotide metabolism	Feeding	Increased
620	ATP-dependent RNA helicase DDX3Y	Ddx3y	Nucleus, cytosol	Nucleotide metabolism	Feeding	Increased
558 + 537	Dihydropyrimidinase-related protein 2	Dpysl2	Cytoskeleton, cytosol	Synapse and trafficking	Feeding	Increased
537	Dihydropyrimidinase-related protein 3	Dpysl3	Cytoplasm	Synapse and trafficking	Feeding	Increased
537	Cytoplasmic dynein 1 light intermediate chain 1	Dync1li1	Cytoskeleton, cytosol	Synapse and trafficking	Feeding	Increased
544	EH domain-containing protein 1	Ehd1	Membrane, endosome	Synapse and trafficking	Feeding	Decreased
42	Fatty acid-binding protein, brain	Fabp7	Cytoplasm	Lipid transport	Feeding	Decreased
493	Peptidyl-prolyl cis-trans isomerase FKBP4	Fkbp4	Cytoplasm, mitochondrion, nucleus	Protein metabolism	Feeding	Decreased
537	Glucose-6-phosphate 1-dehydrogenase X	G6pdx	Cytosol, nucleus	Carbohydrate metabolism	Feeding	Increased
208	Glutamate–cysteine ligase regulatory subunit	Gclm	Cytosol	Glutathione metabolism	Feeding	Decreased
1,100	Glutamine synthetase	Glul	Cytoplasm (mainly)	Amino acid metabolism	Feeding	Increased
318	Guanine nucleotide-binding protein G(o) subunit alpha	Gnao1	Plasma membrane	Synapse and trafficking	Feeding	Decreased
29	Histone H4	Hist1h4a	Nucleus	Chromosome stability	Feeding	Increased
741	Endoplasmin	Hsp90b1	Endoplasmic reticulum	Protein metabolism	Feeding	Decreased
583	Heat shock-related 70 kDa protein 2	Hspa2	Cytoplasm, Cytoskeleton	Protein metabolism	Feeding	Decreased
771	Heat shock 70 kDa protein 4	Hspa4	Cytoplasm	Protein metabolism	Feeding	Decreased
583	Heat shock cognate 71 kDa protein	Hspa8	Membrane, nucleus	Protein metabolism	Feeding	Decreased
583	Stress-70 protein, mitochondrial	Hspa9	Mitochondrion, nucleus	Mitochondrial function	Feeding	Decreased
517	60 kDa heat shock protein, mitochondrial	Hspd1	Mitochondrion	Mitochondrial function	Feeding	Increased
680 + 681	MICOS complex subunit Mic60	Immt	Mitochondrion	Mitochondrial function	Feeding	Increased
517	Alpha-internexin	Ina	Cytoskeleton	Synapse and trafficking	Feeding	Increased
279	LIM and SH3 domain protein 1	Lasp1	Cytoplasm, cytoskeleton	Synapse and trafficking	Feeding	Increased
843	Leucine-rich PPR motif-containing protein, mitochondrial	Lrpprc	Mitochondrion, nucleus	Protein metabolism	Feeding	Increased
639	NADH-ubiquinone oxidoreductase 75 kDa subunit, mitochondrial	Ndufs1	Mitochondrion	Mitoch. function, Energy metab.	Feeding	Decreased
630 + 625 + 620	Vesicle-fusing ATPase	Nsf	Cytosol, Golgi apparatus	Synapse and trafficking	Feeding	Increased
245	Ubiquitin thioesterase OTUB1	Otub1	Cytoplasm	Protein metabolism	Feeding	Decreased
843	Pyruvate carboxylase, mitochondrial	Pc	Mitochondrion	Mitoch. function, carbohydrate metabolism	Feeding	Increased
392 + 1,100	Pyruvate dehyd. E1 component subunit alpha, mitochondrial	Pdha1	Mitochondrion	Mitoch. function, Energy metab.	Feeding	Increased
537	Protein disulfide-isomerase A3	Pdia3	Endoplasmic reticulum	Protein metabolism	Feeding	Increased
208	Prohibitin	Phb	Mitochondrion, nucleus, membrane	Mitochondrial function	Feeding	Decreased
1,244	DNA-directed RNA polymerase II subunit RPB3	Polr2c	Nucleus	Nucleotide metabolism	Feeding	Decreased
279	Serine/threonine-protein phosphatase PP1-beta catalytic subunit	Ppp1cb	Cytoplasm, nucleus	Miscellaneous	Feeding	Increased
279	Serine/threonine-protein phosphatase PP1-gamma catalytic subunit	Ppp1cc	Cytoplasm, mitochondrion, nucleus	Miscellaneous	Feeding	Increased
202	Proteasome subunit beta type-7	Psmb7	nucleus, cytosol	Protein metabolism	Feeding	Decreased
537	Serine–tRNA ligase, cytoplasmic	Sars	Cytoplasm, nucleus	Protein metabolism	Feeding	Increased
339	Neuronal-specific septin-3	Sept3	Cytoskeleton	Synapse and trafficking	Feeding	Increased
493	Septin-4	Sept4	Cytoplasm, cytoskeleton	Synapse and trafficking	Feeding	Decreased
1,023	Septin-5	Sept5	Cytoskeleton	Synapse and trafficking	Feeding	Increased
1,091	Leukocyte elastase inhibitor A	Serpinb1a	Cytoplasm	Protein metabolism	Feeding	Decreased
318	Endophilin-A1	Sh3gl2	Endosome	Synapse and trafficking	Feeding	Decreased
594 + 600	Calcium-binding mitochondrial carrier protein Aralar1	Slc25a12	Mitochondrion	Mitochondrial function	Feeding	Increased
453	Tubulin alpha-1B chain	Tuba1b	Cytoskeleton	Synapse and trafficking	Feeding	Decreased
453	Tubulin beta-3 chain	Tubb3	Cytoskeleton	Synapse and trafficking	Feeding	Decreased
453	Tubulin beta-4A chain	Tubb4a	Cytoskeleton	Synapse and trafficking	Feeding	Decreased
453	Tubulin beta-4B chain	Tubb4b	Cytoskeleton	Synapse and trafficking	Feeding	Decreased
453	Tubulin beta-5 chain	Tubb5	Cytoskeleton	Synapse and trafficking	Feeding	Decreased
1,100	Elongation factor Tu, mitochondrial	Tufm	Mitochondrion	Protein and nucleotide metab.	Feeding	Increased
382	Alpha-centractin	Actr1a	Cytoskeleton	Synapse and trafficking	Feeding + Interaction	Increased + RF > AL ZT5
382	Beta-centractin	Actr1b	Cytoskeleton	Synapse and trafficking	Feeding + Interaction	Increased + RF > AL ZT5
382 + 392	Glutamine synthetase	Glul	Cytoplasm (mainly)	Amino acid metabolism	Feeding + Interaction	Increased + RF > AL ZT5
382	Phosphoglycerate kinase 1	Pgk1	Cytoplasm	Carbohydrate metabolism	Feeding + Interaction	Increased + RF > AL ZT5
541	Protein disulfide-isomerase A3	Pdia3	Endoplasmic reticulum	Protein metabolism	Feeding + Time	Decreased + ZT5 < ZT17
610	ATP synthase subunit beta, mitochondrial	Atp5f1b	Mitochondrion	Mitoch. function, Energy metab.	Feeding + Time	Decreased + ZT5 < ZT17
1,085 + 1,087	Heat shock protein HSP 90-alpha	Hsp90aa1	Membrane, nucleus	Protein metabolism	Feeding + Time	Decreased + ZT5 < ZT17
1,085 + 1,087	Heat shock protein HSP 90-beta	Hsp90ab1	Membrane, nucleus	Protein metabolism	Feeding + Time	Decreased + ZT5 < ZT17
610	Endoplasmic reticulum chaperone BiP	Hspa5	Endoplasmic reticulum	Protein metabolism	Feeding + Time	Decreased + ZT5 < ZT17
541	T-complex protein 1 subunit epsilon	Cct5	Cytoskeleton, cytoplasm	Protein metabolism	Feeding + Time	Decreased + ZT5 < ZT17
610	Spectrin alpha chain, non-erythrocytic 1	Sptan1	Cytoplasm, Cytoskeleton	Synapse and trafficking	Feeding + Time	Decreased + ZT5 < ZT17
53	Ubiquitin-conjugating enzyme E2 N	Ube2n	Nucleus	Protein metabolism	Interaction	RF > AL ZT5 + RF < AL ZT17
82	DNA-directed RNA polymerases I, II, and III subunit RPABC3	Polr2h	Nucleus	Nucleotide metabolism	Interaction	RF > AL ZT5 + RF < AL ZT17
82	Beta-synuclein	Sncb	Cytoplasm, synapse	Synapse and trafficking	Interaction	RF > AL ZT5 + RF < AL ZT17
105	Visinin-like protein 1	Vsnl1	Cytosol	Synapse and trafficking	Interaction	RF > AL ZT5 + RF < AL ZT17
139	Proteasome subunit beta type-4	Psmb4	Nucleus, cytosol	Protein metabolism	Interaction	RF > AL ZT5
155	Ras-related protein Rab-11B	Rab11b	Synapse, endosome	Synapse and trafficking	Interaction	RF < AL ZT5
155	Ras-related protein Rab-5C	Rab5c	Plasma membrane, endosome	Synapse and trafficking	Interaction	RF < AL ZT5
216	Eukaryotic translation initiation factor 4H	Eif4h	Cytoplasm	Protein metabolism	Interaction	RF > AL ZT5
219	Proteasome subunit alpha type-1	Psma1	Nucleus, cytosol	Protein metabolism	Interaction	RF > AL ZT17
317	Guanine nucleotide-binding protein G(o) subunit alpha	Gnao1	Plasma membrane	Synapse and trafficking	Interaction	
317	Endophilin-A1	Sh3gl2	Endosome	Synapse and trafficking	Interaction	
523	Protein disulfide-isomerase A3	Pdia3	Endoplasmic reticulum	Protein metabolism	Interaction	RF < AL ZT5
523	Serine/threonine-protein phosphatase 5	Ppp5c	Plasma membrane, cytoplasm, nucleus	Miscellaneous	Interaction	RF < AL ZT5
554	Heterogeneous nuclear ribonucleoprotein K	Hnrnpk	Cytoplasm, nucleus	Protein and nucleotide metab.	Interaction	
575	Dihydropyrimidinase-related protein 2	Dpysl2	Cytoskeleton, cytosol	Synapse and trafficking	Interaction	ZT5 > ZT17 RF
1,019 + 1,022	Hemoglobin subunit beta-1	Hbb-b1	[Red blood cells]	Miscellaneous	Interaction	ZT5 > ZT17 RF
36	Parvalbumin alpha	Pvalb	Cytosol, nucleus	Synapse and trafficking	Time + Interaction	ZT5 < ZT17 AL, RF intermediate
553	Heterogeneous nuclear ribonucleoprotein K	Hnrnpk	Cytoplasm, nucleus	Protein and nucleotide metab.	Time + Interaction	ZT5 > ZT17 RF
158	Glutamine amidotransferase-like class 1 domain-containing protein 3A, mitochondrial	Gatd3a	Mitochondrion	Mitochondrial function	Time-of-day	ZT5 < ZT17
158	Glutathione S-transferase Mu 1	Gstm1	Cytoplasm	Glutathione metabolism	Time-of-day	ZT5 < ZT17
158	Protein-L-isoaspartate(D-aspartate) O-methyltransferase	Pcmt1	Cytoplasm	Protein metabolism	Time-of-day	ZT5 < ZT17
512	Glucose-6-phosphate 1-dehydrogenase X	G6pdx	Cytosol, nucleus	Carbohydrate metabolism	Time-of-day	ZT5 > ZT17
512	Synapsin-2	Syn2	Cytoskeleton, nucleus	Synapse and trafficking	Time-of-day	ZT5 > ZT17
550	Heterogeneous nuclear ribonucleoprotein K	Hnrnpk	Cytoplasm, nucleus	Nucleotide and Protein metabolism	Time-of-day	ZT5 > ZT17
1,020	Hemoglobin subunit beta-1	Hbb-b1	[Red blood cells]	Miscellaneous	Time-of-day	ZT5 > ZT17
1,218	Vimentin	Vim	Cytoskeleton, nucleus, endoplasmic reticulum, mitochondria	Synapse and trafficking	Time-of-day	ZT5 > ZT17
1,331	Glutamate dehydrogenase 1, mitochondrial	Glud1	Mitochondrion	Mitochondrial function	Time-of-day	ZT5 < ZT17
1,330 + 1,331	ATP synthase subunit alpha, mitochondrial	Atp5f1a	Mitochondrion	Mitoch. function, Energy metab.	Time-of-day	ZT5 < ZT17

*Listed are the proteins identified in the proteins spots of which intensity changes between mouse groups highlighted significant effects of feeding conditions [restricted feeding (RF) vs control conditions: food ad libitum + nighttime feeding on day of sampling (AL)], time-of-day (Zeitgeber ZT5 and ZT17), and their interactions. For Feeding effects, the way protein abundance was changed in RF vs AL is given; for time-of-day and interaction effects, the groups that were different are specified in the right column. Subcellular location is based on annotations from the UnitProtKB database.*

### Effects of Feeding Conditions on Cerebellar Proteins

**FIGURE 4:**
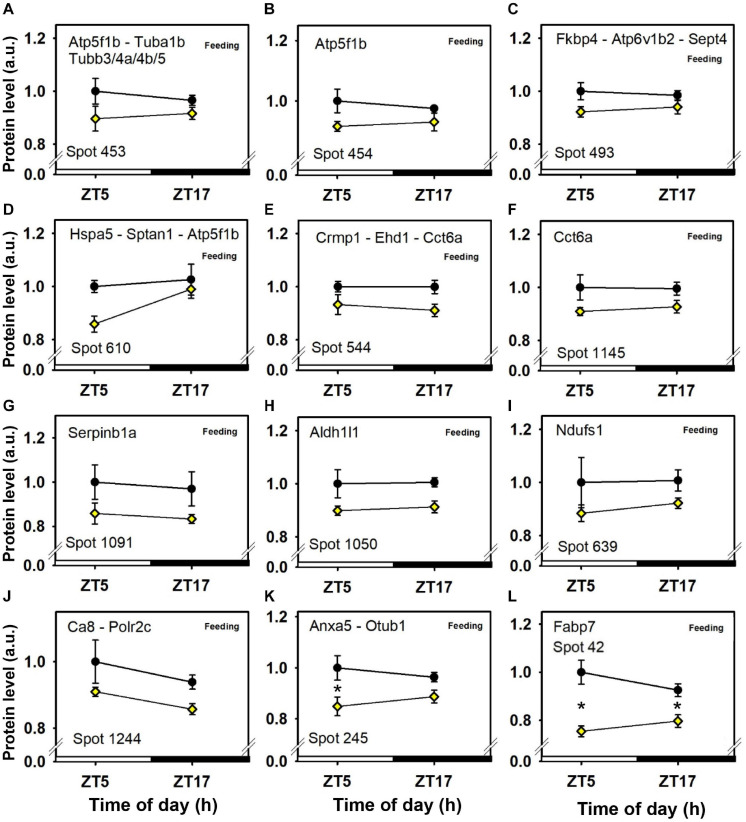
Day–night expression of cerebellar proteins mostly affected by feeding (1/3). From analyses in control mice fed *ad libitum* (closed circles) and mice exposed to daytime restricted feeding (yellow diamonds), “Feeding” indicates significant (*P* < 0.05) main effect of feeding conditions, as detected by two-way ANOVA (see section “Materials and Methods” for details). *P < 0.05 after *post hoc* Tukey comparison. Different part labels have been attributed to each differentially-expressed proteins; see details in the main text.

**FIGURE 5:**
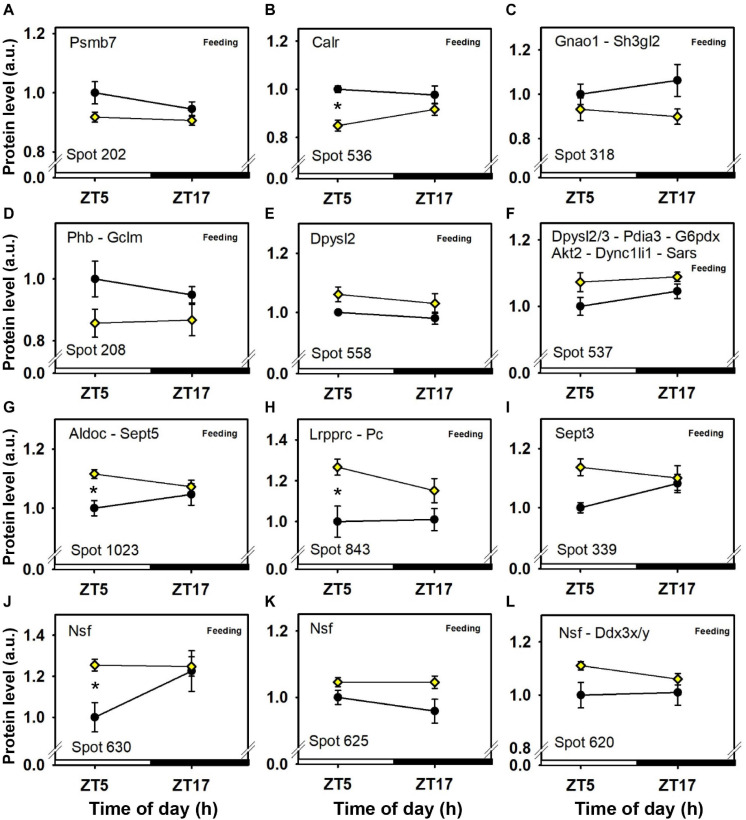
Day–night expression of cerebellar proteins mostly affected by feeding (2/3). From analyses in control mice fed *ad libitum* (closed circles) and mice exposed to daytime restricted feeding (yellow diamonds), “Feeding” indicates significant (*P* < 0.05) main effect of feeding conditions, as detected by two-way ANOVA (see section “Materials and Methods” for details). *P < 0.05 after *post hoc* Tukey comparison. Different part labels have been attributed to each differentially-expressed proteins; see details in the main text.

**FIGURE 6:**
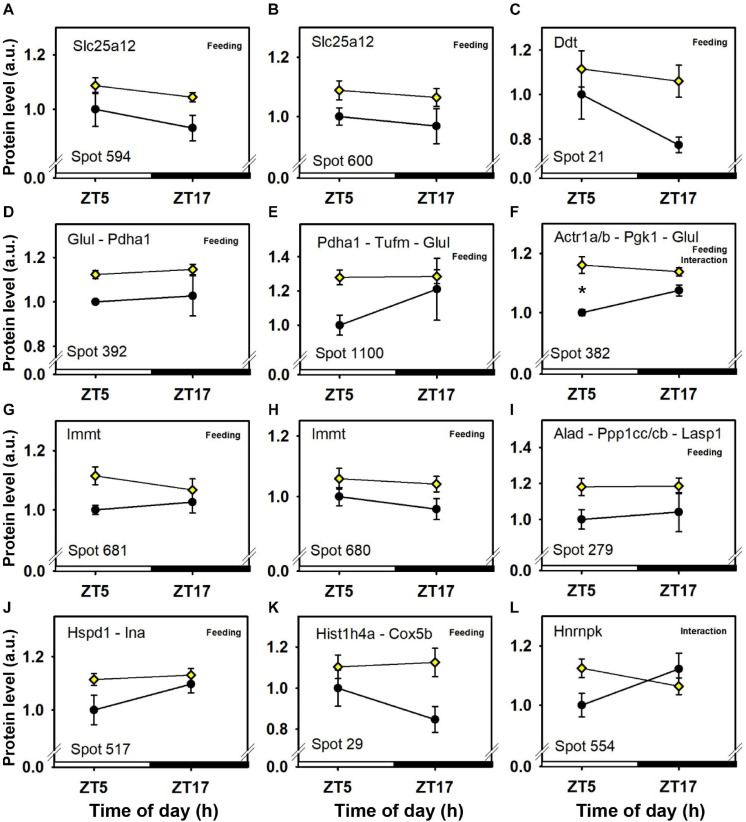
Day–night expression of cerebellar proteins mostly affected by feeding (3/3). From analyses in control mice fed *ad libitum* (closed circles) and mice exposed to daytime restricted feeding (yellow diamonds), “Feeding” and “Interaction” indicate significant (*P* < 0.05) main effects of feeding conditions and time-of-day × feeding interactions, respectively, as detected by two-way ANOVA (see section “Materials and Methods” for details). *P < 0.05 after *post hoc* Tukey comparison. Different part labels have been attributed to each differentially-expressed proteins; see details in the main text.

The intensity of a majority of cerebellar protein spots (37) was changed according to feeding conditions, independently of time-of-day. Three additional spots were decreased by feeding and also affected by time-of-day (Figures 3, 4, 5, 6 and Table 1).

Daytime restricted feeding led to down-regulated levels of a number of heat shock proteins and other chaperones, including heat shock-related 70 kDa protein 2 (Hspa2; spot 583; Figure 3N), heat shock 70 kDa protein 4 (Hspa4; spot 771; Figure 3O), heat shock cognate 71 kDa protein (Hspa8; spot 583; Figure 3N), stress-70 protein (Hspa9; spot 583; Figure 3N), endoplasmin (Hsp90b1; spot 741; Figure 3M), heat shock proteins HSP 90-alpha and -beta (Hsp90aa1 and Hsp90ab1, respectively; spots 1,085 and 1,087; Figures 3K,L), endoplasmic reticulum chaperone BiP (Hspa5; spot 610; Figure 4D), and calreticulin (Calr; spot 536; Figure 5B). The main decrease of Hsp90aa1 and Hspa5 protein contents detected with 2D-DIGE was confirmed by Western blots (Figure 8). Minor differences in statistical results from these two quantitative approaches might be because Western-blotting is not able to detect separately post-translationally modified protein isoforms as 2D-DIGE does (see below). In the category of heat shock proteins, 60 kDa heat shock protein (Hspd1; spot 517; Figure 6J) in the cerebellum was the only one to display increased levels in response to daytime restricted feeding. This was also the case for protein disulfide-isomerase A3 (Pdia3; spot 541; Figure 3J), a protein involved in the response to endoplasmic reticulum stress. Besides heat-shock and stress-related proteins, expression of brain fatty acid binding protein 7 (Fabp7; spot 42. Figure 4L), a protein expressed in astrocytes and neural progenitors that supplies them with fatty acids, was overall down-regulated by restricted feeding.

Abundance of two inner membrane proteins of the mitochondria, namely mitofilin, or MICOS complex subunit Mic60 (Immt; spots 680 and 681; Figures 6G,H) and calcium-binding mitochondrial carrier protein Aralar1 (Slc25a12; spots 594 and 600; Figures 6A,B), is increased by daytime restricted feeding. Furthermore, an increase for cytochrome c oxidase subunit 5B (Cox5b; spot 29; Figure 6K), an enzyme involved in oxidative phosphorylation (OXPHOS) was also observed during daytime restricted feeding. By contrast, mitochondrial NADH-ubiquinone oxidoreductase (Ndufs1; spot 639; Figure 4I) and ATP synthase subunit beta (Atp5f1b; spots 453 and 610; Figures 4A,D), two other OXPHOS enzymes, were less expressed in the cerebellum of daytime food-restricted mice compared to mice fed at night.

In addition, expression levels of several enzymes involved in carbohydrate metabolism, such as fructose-bisphosphate aldolase C (Aldoc; spot 1,023; Figure 5G), pyruvate carboxylase (Pc; spot 843; Figure 6H), phosphoglycerate kinase (Pgk1; spot 382; Figure 6H), and pyruvate dehydrogenase E1 component subunit alpha (Pdha1; spots 392 and 1,100; Figures 6D,E), were higher in the cerebellum during daytime restricted feeding. Other cerebellar proteins were also more expressed during daytime restricted feeding, such as Histone 4 (Hist1h4; spot 29; Figure 6K), vesicle-fusing ATPase (Nsf; spots 620, 625 and 630; Figures 5J–L), PP1-gamma and -beta catalytic subunits (Ppp1cc and Ppp1cb, respectively; spot 279; Figure 6I), T-complex protein 1 subunit epsilon (Cct5; spot 541; Figure 3J), mitochondrial elongation factor Tu (Tufm; spot 1,100; Figure 6E), ATP-dependent RNA helicases (Ddx3x and Ddx3y; spot 620; Figure 5L), mitochondrial leucine-rich PPR motif-containing protein (Lrpprc; spot 843; Figure 5H), and serine-tRNA ligase (Sars; spot 537; Figure 5F). On the other hand, several cerebellar proteins were less expressed in response to daytime restricted feeding, including carbonic anhydrase-related protein (Ca8; spot 1,244; Figure 5G), Cytosolic 10-formyltetrahydrofolate dehydrogenase (Aldh1l1; spot 1,050; Figure 4H), Annexin A5 (Anxa5; spot 245; Figure 4K), T-complex protein 1 subunit zeta (Cct6a; spots 544 and 1,145; Figures 4E,F), ubiquitin thioesterase (Otub1; spot 245; Figure 4K) and proteasome subunit beta type-7 (Psmb7; spot 202; Figure 5A).

A preliminary analysis of clock proteins in the cerebellum suggests that PER2 and BMAL1 levels in mice fed *ad libitum* may, respectively, increase and decrease between noon and midnight (Supplementary Figure 3). These trends fit with the daily patterns of clock genes found in the cerebellum of rats and mice (Mendoza et al., 2010; Rath et al., 2012). In response to daytime restricted feeding, cerebellar levels of both PER2 and BMAL1 appear to be dampened and shifted (Supplementary Figure 3), in accordance with previous findings of clock gene expression (Mendoza et al., 2010).

### Feeding × Time-of-Day Interactions on Cerebellar Proteins

**FIGURE 7:**
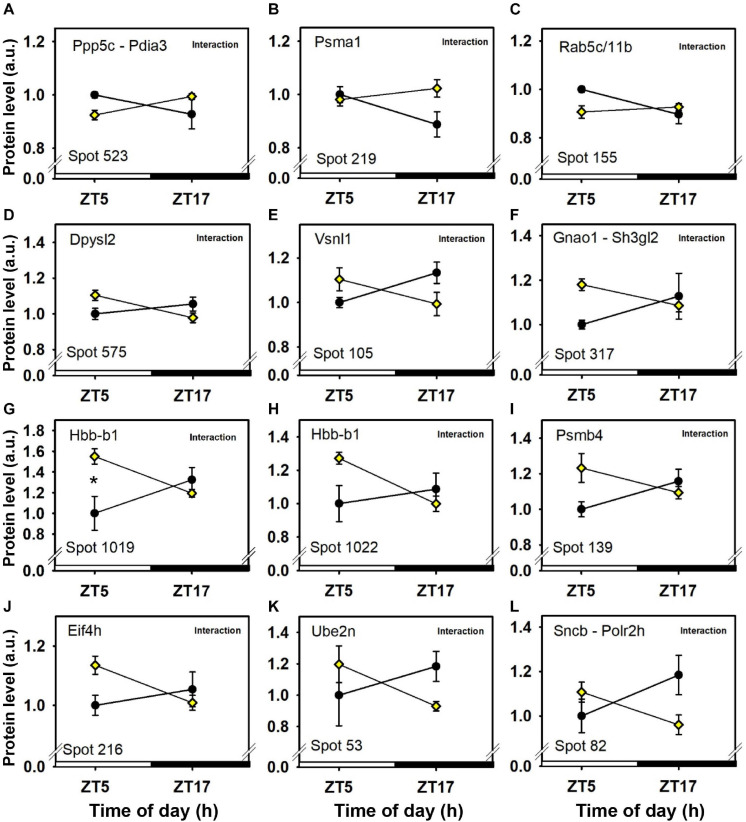
Day–night expression of cerebellar proteins mostly affected by interactions between feeding conditions and time-of day. From analyses in control mice fed *ad libitum* (closed circles) and mice exposed to daytime restricted feeding (yellow diamonds), “Interaction” indicates significant (*P* < 0.05) time-of-day × feeding interaction, respectively, as detected by two-way ANOVA (see section “Materials and Methods” for details). *P < 0.05 after *post hoc* Tukey comparison. Different part labels have been attributed to each differentially-expressed proteins; see details in the main text.

Significant feeding × time-of-day interactions without main effects of either feeding conditions or time-of-day were found for proteins identified in 13 protein spots (Figures 6, 7 and Table 1). Furthermore, for three additional protein spots, significant interactions between feeding and time-of-day were also detected together with a main effect of either time-of-day or feeding (Figures 3, 6 and Table 1).

**FIGURE 8:**
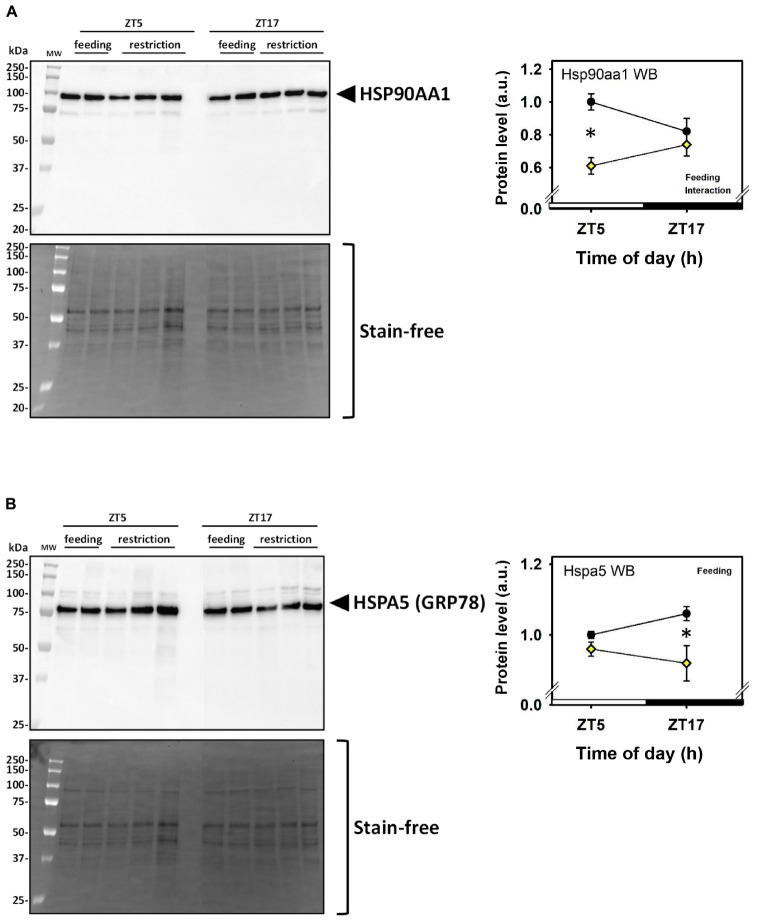
Day–night expression of cerebellar proteins affected by feeding assessed by Western blots. **(A)** Representative Western blot (WB; left panel) and mean protein levels (right panel) for Hsp90aa1, to be compared with spot 1,085 of 2D-DIGE analysis (spots 1,085 and 1,087, see Figure 2). **(B)** Representative Western blot (WB; left panel) and mean protein levels (right panel) for Hspa5, to be compared with spot 1,085 of 2D-DIGE analysis (spot 610, see Figure 3). In the right panels, Feeding and Interaction indicate significant (*P* < 0.05) main effects of feeding conditions or time-of-day × feeding interaction, respectively, as detected by two-way ANOVA (see section “Materials and Methods” for details). *P < 0.05 after *post hoc* Tukey comparison.

A number of cerebellar proteins were more expressed before food access in daytime-fed mice, including glutamine synthetase (Glul; spot 382; Figure 6F; see also spots 392 and 1,100; Figures 6D,E), parvalbumin (Pvalb; spot 36; Figure 3H), visinin-like protein 1 (Vsnl1; spot 105; Figure 7E), guanine nucleotide-binding protein G(o) subunit alpha (Gnao1; spot 317; Figure 7F) and heterogeneous nuclear ribonucleoprotein K (Hnrnpk; spots 553 and 554; Figures 3I, 6L). By contrast, very few cerebellar proteins were specifically less expressed before food access in daytime-fed mice, such as Serine/threonine-protein phosphatase 5 (Ppp5c; spot 523; Figure 7A) and Ras-related protein Rab-5C and 11B (Rab5c and rab11b, respectively; spot 155; Figure 7C).

## Discussion

To determine which cerebellar proteins are modified by time-of-day and/or feeding time, this study investigated the day–night variations of cerebellar proteome in daytime-fed mice compared to control animals fed during nighttime. Among the 89 identified proteins that exhibited statistically changed abundances in the mouse cerebellum, a majority (i.e., 60) was affected only by feeding conditions, reflecting changes in homeostatic processes during temporal food shortage, while very few cerebellar proteins (i.e., 10) were modulated exclusively by daily cues. Several changes in protein abundance (i.e., 16) detected by statistical interactions in daytime food-restricted mice occurred specifically around midday (i.e., before food access).

### Limitations of the Study

Several limitations of the present study can be mentioned. First, the four studied groups of mice were not all fed and fasted for the same durations. On the day of sampling, control mice sampled at ZT5 and food-restricted mice sampled at ZT17 had fasted for 5 h since their last meal. By contrast, control mice sampled at ZT17 had been feeding for 5 h, while food-restricted mice sampled at ZT5 had fasted for 17 h since their last meal. Accordingly, differences between these two experimental groups may rely on feeding conditions and/or time-of-day. Secondly, the mild reduction of calorie intake in food-restricted mice and their body mass loss may have contributed to the effects of feeding conditions. Thirdly, the lack of sizeable differences in clock proteins between the studied groups suggests that the number of differentially expressed proteins identified in the current study may be underestimated. Fourthly, because only the cerebellum was investigated, it cannot be concluded yet whether the observed effects are specific to that structure or if they would be generally detectable in other brain areas.

### Effects of Time-of-Day on Cerebellar Proteins

A number of cerebellar proteins implicated in intracellular metabolism displayed reduced levels at midday compared to midnight, such as one redox regulator (glutathione S-transferase Mu 1, Gstm1) and mitochondrial proteins involved, among other functions, in the metabolism of glutamine (glutamate dehydrogenase 1, Glud1; glutamine amidotransferase-like class 1 domain-containing protein 3A, Gatd3a), in cellular energy homeostasis (ATP synthase subunit alpha, Atp5f1a), and in protein repair [protein-L-isoaspartate(D-aspartate) O-methyl-transferase, Pcmt1]. By contrast, other metabolic proteins displayed higher levels at midday compared to midnight, such as those of the pentose phosphate pathway (i.e., glucose-6-phosphate 1-dehydrogenase X, G6pdx). In addition, proteins involved in synapse activity (Synapsin-2, Syn2), cytoskeleton (Vimentin, Vim), and pre-mRNA processing (heterogeneous nuclear ribonucleoprotein K, Hnrnpk) were also increased at midday. The protein spots whose intensity was only affected by time-of-day highlight proteins or protein isoforms that are not modified by timing of food availability. This suggests that these proteins or protein isoforms are not directly controlled by the cerebellar clock, which is shifted according to daily access to food (Mendoza et al., 2010). The day–night variations in above reported proteins may be due to extra-cerebellar influence. For instance, the master clock in the suprachiasmatic nuclei, known to be mostly insensitive to meal time, may provide similar temporal cues in mice fed *ad libitum* and in those challenged with daytime restricted feeding. In accordance with this possibility, a previous study has shown an impact of suprachiasmatic lesions on cerebellar rhythmicity (Rath et al., 2012). Alternatively, day–night variations in cerebellar protein abundances without an effect due to restricted feeding may denote a similar influence of ambient light and/or darkness cues in both groups of animals.

Post-translational modifications of proteins generally alter protein activity levels and/or half-lives. Because phosphorylation and acetylation trigger changes in protein isoelectric point, protein variants due to such modifications can be segregated from native proteins in different protein spots using two-dimensional electrophoresis (Scumaci and Cuda, 2019). Here, G6pdx has been identified in two different protein spots, one affected by time-of-day only (see above), and another one affected by feeding conditions only (see below). In addition, Hnrnpk has been identified in three protein spots, one affected by time-of-day only (see above), a second one by both time-of-day and the interaction with feeding conditions, and a third one by the interaction only (see below). According to the Swissprot database (accessed in September 2020), G6pdx has one phosphorylation site and one acetylation site, while Hnrnpk has multiple phosphorylation and acetylation sites, and these modifications may impact activity of these two proteins. Hence, the various isoforms of these two proteins differ in their responses to time-of-day and feeding conditions. Post-translational modifications of proteins therefore appear involved in the response of the cerebellar proteome to schedule changes in the availability of food over the day. Unfortunately, our protocol was not dedicated to characterize post-translational modifications.

### Effects of Feeding Conditions on Cerebellar Proteins

In total, 65 different proteins were either down- or up-regulated by temporal restricted feeding, such a high number indicating that the feeding challenge we used triggers specific effects, and not simply a general reduction of protein synthesis. The targeted changes observed rather reflect specific modifications of homeostatic processes associated with reduced food availability and moderate reduction of body mass.

It is noteworthy that independently of time-of day, a number of heat-shock proteins and closely related proteins were down-regulated by restricted feeding, such as heat shock-related 70 kDa protein 2 (Hspa2), heat shock 70 kDa protein 4 (Hspa4), endoplasmic reticulum chaperone BiP (Hspa5), heat shock cognate 71 kDa protein (Hspa8), stress-70 protein (Hspa9, endoplasmin (Hsp90b1), heat shock proteins HSP 90-alpha and -beta (Hsp90aa1 and Hsp90ab1, respectively), and calreticulin (Calr), a peptide binding heat shock proteins. In sharp contrast, expression of heat shock proteins in the rat hypothalamus, including suprachiasmatic nuclei, is increased by various cellular stressors, such as heat stress and calorie restriction (Aly et al., 1994; Goh et al., 2016). In our study, only 60 kDa heat shock protein (Hspd1) and protein disulfide-isomerase A3 (Pdia3) displayed an increased cerebellar expression in response to restricted feeding. The present data thus suggest distinct homeostatic regulation of heat shock proteins during food shortage in the cerebellum compared to hypothalamus. Several of these chaperones are involved in the correct folding of newly synthetized proteins (Calr), the unfolded protein response (Hspa5, Hsp90b1), and endoplasmic reticulum-associated degradation (Hspa2, Hspa8, Hsp90aa1 and Hsp90ab1) (Naidoo, 2009). Decreased levels of ubiquitin thioesterase (Otub1) and proteasome subunit beta type-7 (Psmb7) could also support lowered endoplasmic reticulum-associated degradation in daytime-fed mice. The regulation of chaperone, HSP, and related protein levels may thus reflect a general trend toward reduced protein folding during energy restriction and/or indicate that endoplasmic reticulum functions are altered. Because the levels of important factors in transcription and aminoacyl-tRNA biosynthesis, namely mitochondrial elongation factor Tu (Tufm), ATP-dependent RNA helicases (Ddx3x and Ddx3y), mitochondrial leucine-rich PPR motif-containing protein (Lrpprc), and serine-tRNA ligase (Sars), were higher in the cerebellum of daytime restricted mice, it is unlikely that alteration of protein folding is accompanied by a general decrease in protein synthesis. Nevertheless, in support of altered protein folding during daytime feeding restriction compared to control nighttime access to food, levels of two components of the chaperonin-containing T-complex (Dunn et al., 2001), namely T-complex protein 1 subunits zeta (Cct6a) and epsilon (Cct5), were respectively lower and higher. Finally, HSP90 proteins are repressors of heat shock factor 1 (HSF1), a critical transcription factor in the response to heat stress and other cellular stressors (Zou et al., 1998). HSF1 has also been identified as a circadian transcription factor that interacts with other clock proteins, notably in response to activation of heat shock pathway (Reinke et al., 2008; Tamaru et al., 2011). Further studies will be needed to understand if and how a shifted window of daily feeding can modulate HSF1 action and heat shock pathway in the cerebellum.

Besides heat-shock proteins, among the numerous other down-regulated cerebellar proteins during daytime restricted feeding, there was brain fatty acid binding protein 7 (Fabp7), a protein expressed in astrocytes and neural progenitors that supplies them with fatty acids such as docosahexanoic (DHA) and arachidonic acids. In the cerebellum, rhythmic expression of *Fabp7* mRNA peaks around midday, while Fabp7 protein show higher levels in late afternoon (Gerstner et al., 2008; Plumel et al., 2019). The present findings indicate that the influence of daytime restricted feeding overrides circadian expression of Fabp7 and diminishes its levels, maybe leading to a reduced uptake of fatty acids in astrocytes. Reduced and shifted fatty acid uptake, notably DHA, may then develop into reduced cerebellar levels, possibly modifying cerebellum functioning (Denis et al., 2013).

At the mitochondrial level, two inner membrane proteins, one involved in maintenance of cristae morphology (i.e., mitofilin, or MICOS complex subunit Mic60; Immt) (Ott et al., 2015) and another one involved in calcium-dependent exchange of cytoplasmic glutamate with mitochondrial aspartate (i.e., calcium-binding mitochondrial carrier protein Aralar1; Slc25a12) (Thangaratnarajah et al., 2014), were more expressed during daytime restricted feeding. This indicates that dynamics of mitochondrial structure and function are likely modified by the shifted pattern of energy supply. Furthermore, among enzymes involved in oxidative phosphorylation (OXPHOS), only cytochrome c oxidase subunit 5B (Cox5b) was more expressed in the cerebellum during daytime restricted feeding. Decreased levels of two OXPHOS proteins (i.e., mitochondrial NADH-ubiquinone oxidoreductase, Ndufs1, and ATP-synthase subunit beta, Atp5f1b) during daytime restricted feeding may be indicative of a reduction in metabolic activity of the cerebellum, in particular before food access. In keeping with this hypothesis, local cerebral metabolic rate for glucose measured using 2-[^14^C]-deoxyglucose is reduced in the cerebellar cortex of daytime food-restricted rats prior to time of feeding (de Vasconcelos et al., 2006). However, a certain number of cerebellar proteins involved in carbohydrate metabolism were up-regulated in daytime food-restricted mice. Fructose-bisphosphate aldolase C [Aldoc, also known as zebrin II (ZII]), is an enzyme involved in both glycolysis and gluconeogenesis. Of note Aldoc is heterogeneously expressed in Purkinje cells in which they define well-defined sagittal stripes, either highly expressing or lacking Aldoc (Brochu et al., 1990). Aldoc levels are increased in the liver of old, but not young, mice under long-term calorie restriction (Hagopian et al., 2003). Here, it is noteworthy that Aldoc levels were high in the cerebellum of daytime food-restricted mice, particularly prior to the time of feeding (i.e., ZT5). Such a time-point corresponds to the longest duration of daily fasting among the studied groups, and glycolysis/gluconeogenesis might have reached higher rates in the cerebellum. Accordingly, pyruvate carboxylase (Pc), that plays a role in gluconeogenesis, phosphoglycerate kinase (Pgk1), a glycolytic enzyme, and pyruvate dehydrogenase E1 component subunit alpha (Pdha1), which links glycolysis to the tricarboxylic acid cycle, were more highly expressed in the cerebellum during daytime food restriction, especially before feeding time for Pc and Pgk1. Thus, measurements of glycolytic enzyme activities and/or levels of glycolytic intermediates will be required to better assess how cerebellar glucose and fructose metabolism responds to changes in food availability.

Despite its name, carbonic anhydrase-related protein (Ca8) likely lacks carbonic anhydrase activity. Nevertheless, Ca8 is highly expressed in Purkinje cells in which it regulates dendritic growth (Shimobayashi et al., 2016) and mutated Ca8 leads to cerebellar ataxia (Kaya et al., 2011). Here we show that expression of Ca8 is down-regulated during daytime food restriction, maybe slowing down dendritic growth.

Cytosolic 10-formyltetrahydrofolate dehydrogenase (Aldh1l1), one of the main folate-metabolizing enzyme, was down-regulated in daytime food-restricted mice. Because folate participates in DNA methylation and food restriction affects such epigenetic mechanism (Hernandez-Saavedra et al., 2019; Krupenko and Krupenko, 2019), reduced expression of Aldh1l1 may ultimately lead to decreased levels of methylated DNA that would modify transcription and DNA repair. Histone 4 (Hist1h4) is a protein well-known to be involved in chromatin remodeling (Zocchi and Sassone-Corsi, 2010). Up-regulation of Hist1h4 in the cerebellum of daytime food-restricted mice suggests changes in chromatin compaction state during daytime restricted feeding in that hindbrain structure. Further studies are needed to evaluate these putative epigenetic changes by quantifying folate levels, markers of DNA methylation and histone modifications in the cerebellum of daytime food-restricted mice.

Although its mRNA levels do not oscillate on a daily basis, vesicle-fusing ATPase (Nsf) is a protein rhythmically expressed in the mouse cerebellum of mice fed *ad libitum*, with a daily peak of accumulation around the middle of the night (Plumel et al., 2019). Being present in 3 distinct protein spots, expression of Nsf is clearly up-regulated in the cerebellum of daytime food-restricted mice, possibly reflecting an increased neuronal activity as shown during food expectation by c-Fos activation in the cerebellum (Poulin and Timofeeva, 2008). We already mentioned elsewhere in this paper that several protein isoforms were identified in distinct protein spots and they were found to be regulated in a specific manner among mouse groups. Protein post-translational modification states in the cerebellum of mice therefore appear modulated during energy restriction (Atp5f1b, Cct6a, Dpysl2, G6pdx, Glul, Gnao1, Immt, Nsf, Pdha1, Pd1a3, and Slc25a12). Further investigation would be required to determine to what extent such modifications affect biological processes in response to feeding conditions.

As discussed in our previous proteomic analysis (Plumel et al., 2019), clock proteins in the cerebellum were not detected using 2D-DIGE-MS, probably due to their low levels of expression. Nevertheless, post-translational modifications of clock proteins are among the regulatory mechanisms that drive circadian oscillations (Mauvoisin and Gachon, 2019). Accordingly, serine/threonine-protein phosphatase PP1 has been identified as a strong regulator of circadian clocks, being able among others to phosphorylate the clock protein PER2 (Schmutz et al., 2011). Thus, it is worth highlighting that expression of PP1-gamma and -beta catalytic subunits (Ppp1cc and Ppp1cb, respectively) was up-regulated in the cerebellum of food-restricted mice, raising the possibility that increased phosphorylation of clock proteins participate in the synchronizing effects of restricted feeding upon the cerebellar clock (Mendoza et al., 2010).

### Feeding × Time-of-Day Interactions on Cerebellar Proteins

Significant feeding × time-of-day interactions allow detecting specific changes for a given feeding condition (daytime- or nighttime feeding) close to midday (Zt5) or midnight (ZT17). We propose that the highlighted proteomic differences around midday (i.e., time-point during which food-anticipatory activity is expressed in food-restricted mice) may reveal changes in processes related or concomitant to food anticipation.

In the brain, glutamine synthetase (Glul) converts excess of glutamate together with ammonium into glutamine. This pathway is considered as a buffer against the neurotoxicity of high concentrations of cytoplasmic glutamate. Glul levels which are increased in the cerebellum of food-restricted animals, especially before feeding time, may indicate activation of glutamatergic pathways in cerebellar neurons (granular cells) and/or increased uptake in Bergmann glial cells (Bordey and Sontheimer, 2003) during food anticipation.

Levels of two calcium-binding proteins, namely parvalbumin (Pvalb) and visinin-like protein 1 (Vsnl1), were inversely regulated in the cerebellum of food-restricted mice, as compared to control animals fed at night. These proteins are distributed in a cell-specific manner in the cerebellum, Pvalb and Vsnl1 being localized in Purkinje and granule cells, respectively (Spilker et al., 2002; Bastianelli, 2003). The observed temporal changes in both Pvalb and Vsnl1 suggest a global change in calcium signaling within the cerebellum of restricted mice during food expectation in comparison to late post-prandial and sleep-induced fasting in control fed mice at the same time-of-day.

Expression of guanine nucleotide-binding protein G(o) subunit alpha (Gnao1) is also enhanced in the cerebellum of daytime food-restricted mice only during food expectation (spot 317). Such window of up-regulation may mediate specific activation of Go-coupled transduction pathways in the cerebellum.

Heterogeneous nuclear ribonucleoprotein K (Hnrnpk) is a protein rhythmically expressed in the cerebellum with maximal levels in the middle of the active/feeding period when food is available *ad libitum* (Plumel et al., 2019). D-site binding protein (Dbp) is a clock-controlled transcription factor that also feeds back to the molecular clock via D-boxes (Lopez-Molina et al., 1997; Yamajuku et al., 2011). In the cerebellar cortex, Dbp is rhythmically expressed with an acrophase at the onset of the active period in rats fed *ad libitum* (Rath et al., 2012). Interestingly, Hnrnpk activates the transcription of Dbp and possibly of clock proteins (Kwon et al., 2020). Therefore, it is tempting to speculate that the elevated expression of given isoforms of Hnrnpk in the cerebellum of daytime food-restricted mice prior to meal time (i.e., spot 554) participates to some extent to feeding-induced shift of the cerebellar clockwork.

As aforementioned, post-translational modifications of clock proteins tightly regulate circadian oscillations. Serine/threonine-protein phosphatase 5 (Ppp5c) stimulates the activity of casein kinase I ε, while the clock proteins cryptochrome 1 and 2 inhibit activity of Ppp5c (Partch et al., 2006). Accordingly, it should be noted that Ppp5c is among the rare proteins to be specifically reduced at the time of food anticipation, a down-regulation that may be related to circadian processes. Ras-related protein Rab-5C and 11B (Rab5c and rab11b, respectively), which are regulators of intracellular membrane trafficking, were concomitantly reduced, possibly reflecting slow-down trafficking in the cerebellum of mice anticipating food access.

In conclusion, this study investigated day–night modifications in the cerebellar proteome of mice fed either during 6-h daytime or 12-h nighttime. A majority of identified proteins in the cerebellum was affected only by feeding conditions, thus involving changes in homeostatic processes during limited food availability. Levels of few other cerebellar proteins were modulated exclusively by daily (or circadian) cues, because they were independent of meal time. Finally, a set of cerebellar proteins was changed due to combined influence of meal time and time-of-day, as detected by significant interactions. We propose that these proteins affecting glutamate metabolism (Glul), intracellular calcium (Pvalb, Vsnl1), Go-coupled transduction (Gnao1) and clockwork (Hnrnpk, Ppp5c) may, among other functions, participate in behavioral anticipation of mealtime and/or feeding-induced shift in the circadian clock of the cerebellum. Further investigations will be needed to unravel the respective roles of these targeted regulatory changes within the cerebellum.

## Data Availability Statement

The mass spectrometry proteomics data have been deposited to the ProteomeXchange Consortium via the PRIDE (Vizcaino et al., 2016) partner repository with the dataset identifier PXD021056.

## Ethics Statement

The experiments were performed in accordance with the NIH Guide for the Care and Use of Laboratory Animals (1996), the French National Law (implementing the European Union Directive 2010/63/EU) and approved by the Regional Ethical Committee of Strasbourg for Animal Experimentation (CREMEAS) and French Ministry of Higher Education and Research (APAFIS #2533-2015110215078867 v1).

## Author Contributions

EC conceived the study and carried out the experimental work on mice. FB and MP conducted the proteomic analyses. FB, PM, and AH conducted the Western Blot analyses. EC and FB drafted the manuscript. All authors gave final approval for publication.

## Conflict of Interest

The authors declare that the research was conducted in the absence of any commercial or financial relationships that could be construed as a potential conflict of interest.

## Footnotes

1 http://proline.profiproteomics.fr/
