# Supplementary figures and images for: Daytime Restricted Feeding Affects Day–Night Variations in Mouse Cerebellar Proteome
Source: Front Mol Neurosci. 2021 Apr 12;14:613161. doi: 10.3389/fnmol.2021.613161 (PMC8072461; doi:10.3389/fnmol.2021.613161)

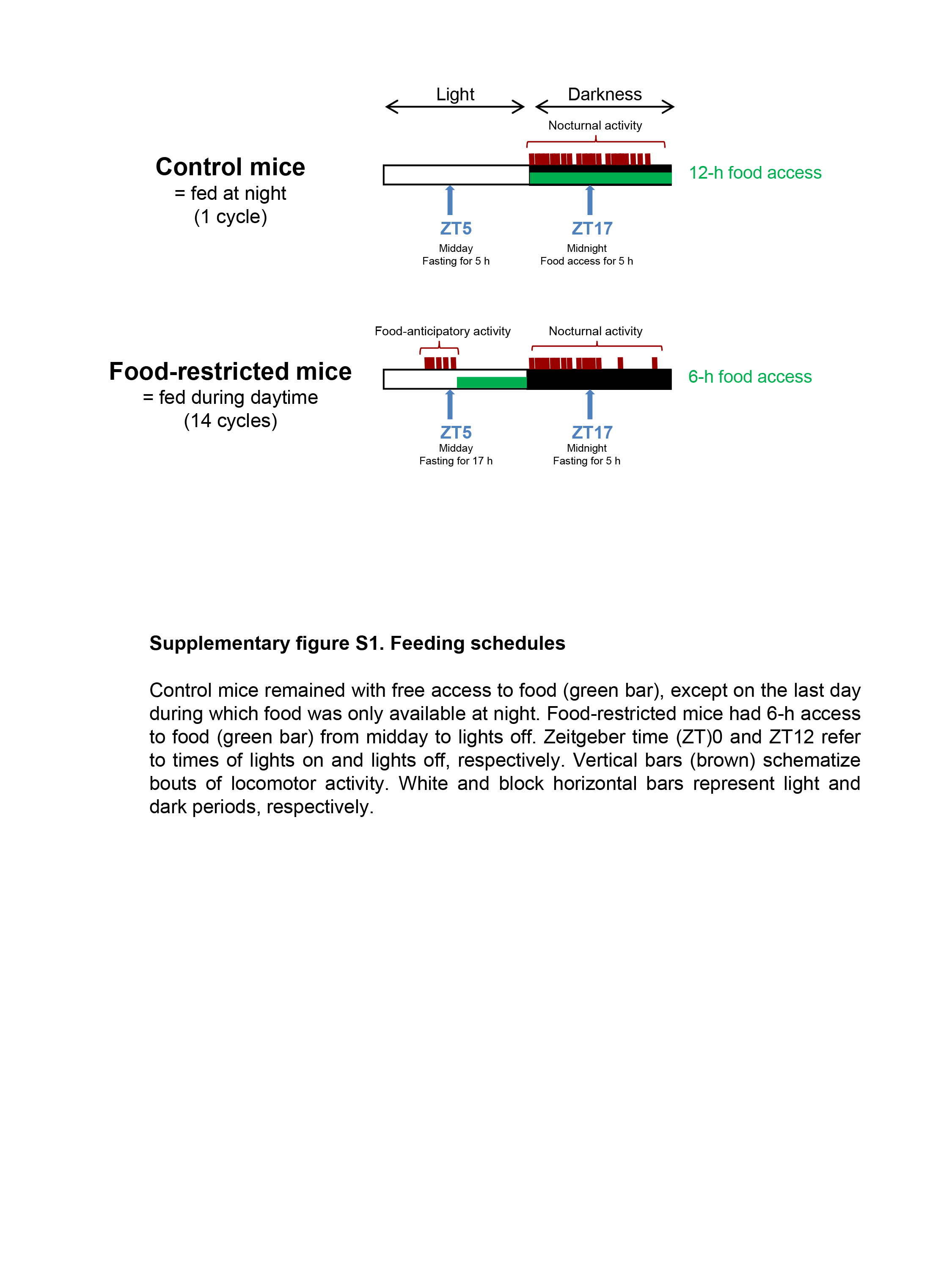

Supplement: Supplementary file 2 [file Image_1.JPEG]

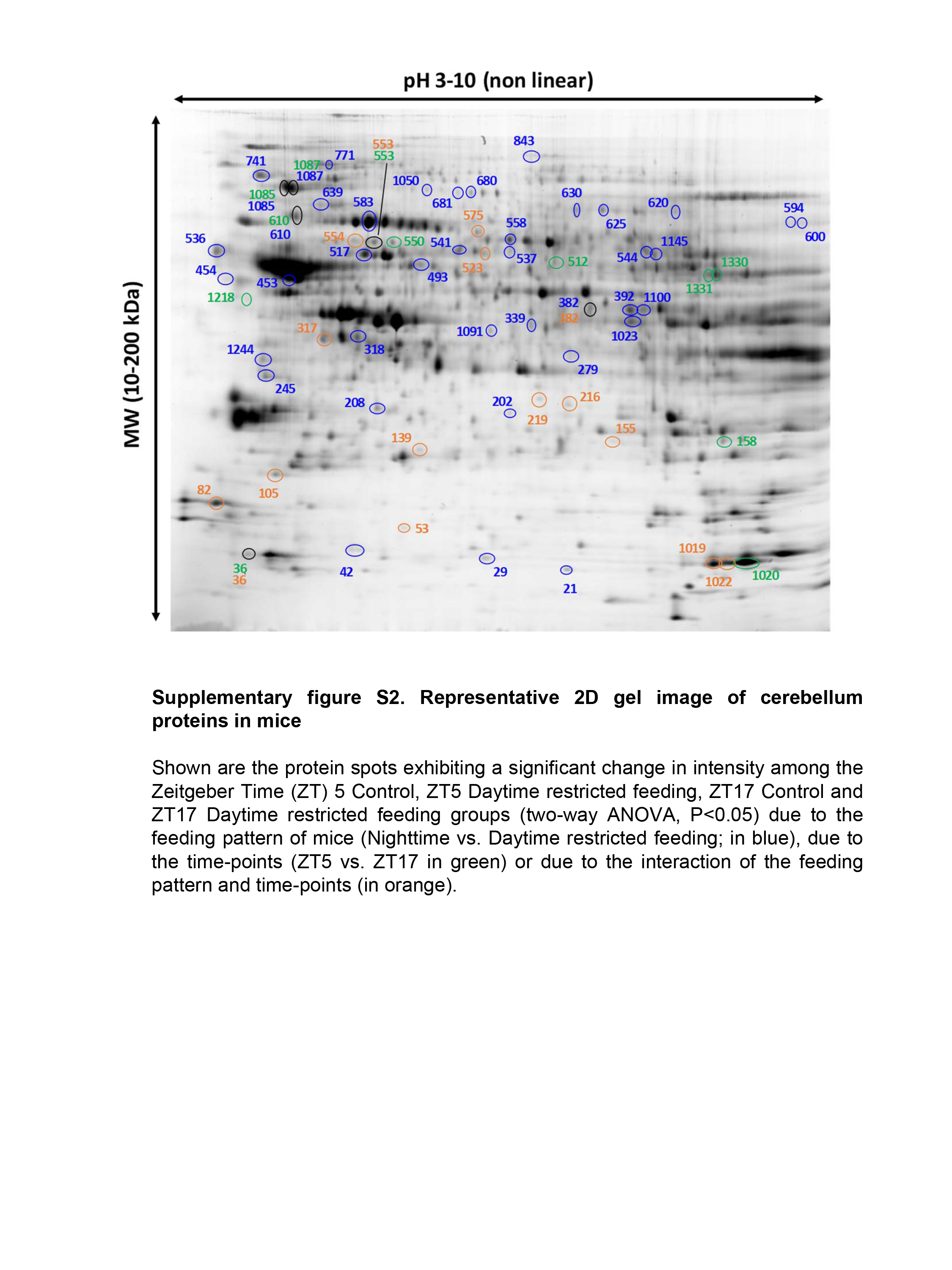

Supplement: Supplementary file 3 [file Image_2.JPEG]

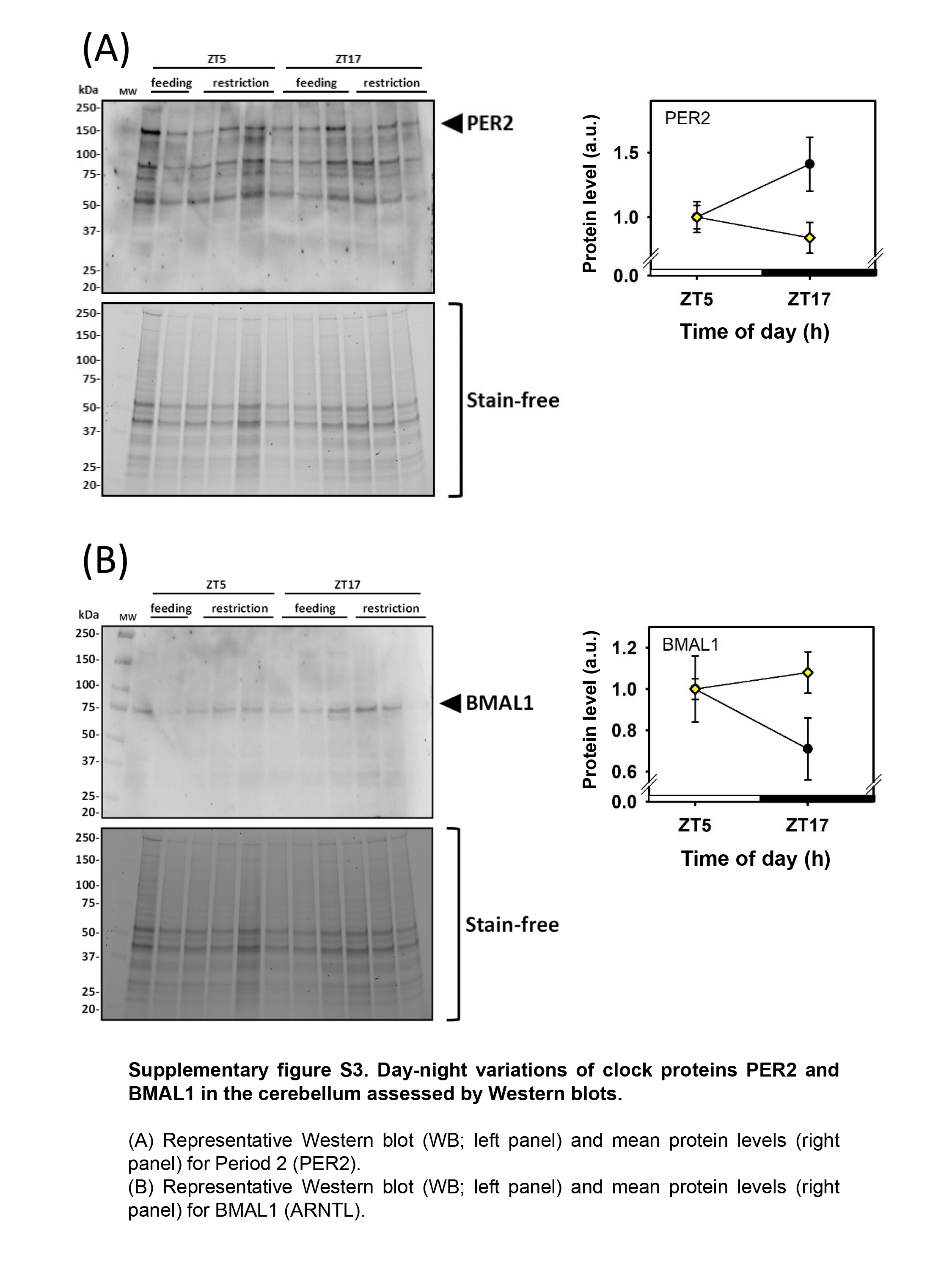

Supplement: Supplementary file 4 [file Image_3.JPEG]
